# The cumulative false-positive rate in colorectal cancer screening: a Markov analysis

**DOI:** 10.1097/MEG.0000000000001669

**Published:** 2020-03-04

**Authors:** Ulrike Haug, Veerle M.H. Coupé

**Affiliations:** a Department of Clinical Epidemiology, Leibniz Institute for Prevention Research and Epidemiology – BIPS; b Faculty of Human and Health Sciences, University of Bremen, Bremen, Germany; c Department of Epidemiology and Biostatistics, Amsterdam Public Health Research Institute, Amsterdam UMC, Vrije Universiteit Amsterdam, Amsterdam, The Netherlands

**Keywords:** colorectal cancer, false-positive, screening

## Abstract

**Background:**

Faecal occult blood testing is widely used in colorectal cancer screening. However, there is little empirical long-term evidence on the accumulation of false-positive test results over several screening rounds. We aimed to systematically explore and quantify the cumulative false-positive rate for various scenarios of colorectal cancer screening.

**Methods:**

Using a Markov analysis, we estimated the lifetime cumulative number of false-positive test results (cumFP) per 100 000 50-year-old persons. We varied the screening interval and the specificity of a single screening test and the starting age of screening.

**Results:**

For a test with a specificity of 98% used from 50 to 74 years, the cumFP at age 74 was 26 260 (1-year interval), 15 102 (2-year interval), and 10 819 (3-year interval), respectively. For a test with a specificity of, respectively, 95 and 92% used at a 2-year interval, the cumFP at age 74 was 2.2 times and 3.0 times higher as compared to a test with a specificity of 98%. The cumFP at age 74 was 18% lower for screening persons aged 54–74 years vs. 50–74 years.

**Conclusion:**

Our findings quantitatively illustrate the large variation of the cumFP in colorectal cancer screening between screening strategies, which is relevant to informed decision making and adequate resource planning.

## Abstract 2

Supplemental Digital Content is available in the text.

## Introduction

Worldwide, colorectal cancer (CRC) is the third most common cancer with more than 1.8 million new cases per year and the second most common cancer cause of death, with more than 800 000 deaths per year [1]. Randomized controlled trials have shown that biennial screening with the guaiac-based faecal occult blood test reduces CRC mortality by 15% [2]. Since the conduct of these trials, new non-invasive screening tests have been developed – such as the faecal immunochemical test (FIT) for haemoglobin or the stool DNA test – that are used or considered to be used in a similar manner [3,4]. This means that the test is repeated at regular intervals and positive test results are followed up by colonoscopy, yielding a certain rate of true-positive and false-positive test results at each screening round.

When considering not only one but a series of screening rounds, that is, taking the longitudinal rather than the cross-sectional perspective, it has been recognized that the true-positive rate increases over rounds given that a lesion missed at one round may be detected at a subsequent round [5]. Accordingly, it was suggested to distinguish between per-test sensitivity and program sensitivity (i.e. sensitivity of a series of tests). Similarly, the false-positive rate accumulates over the screening rounds, that is, a lesion-free person testing negative at one round may test positive at a subsequent round while still being lesion-free. The cumulative false-positive rate is an important feature of a screening program, for example, regarding the patient burden and the resources required for screening. Its consideration is even more relevant for new screening tests that have a high per-test sensitivity, but a relatively low per-test specificity, as it was reported for the stool DNA test [4].

The cumulative false-positive rate depends on several factors: (1) the number of screening rounds which in turn is determined by the screening interval and the screening age range, (2) the per-test specificity, and (3) the conditional (in-)dependence of sequential testing. Taking into account these factors, we aimed to systematically quantify and illustrate the cumulative false-positive rate for various scenarios of non-invasive CRC screening by means of a basic Markov model.

## Methods

### Model structure

**Fig. 1:**
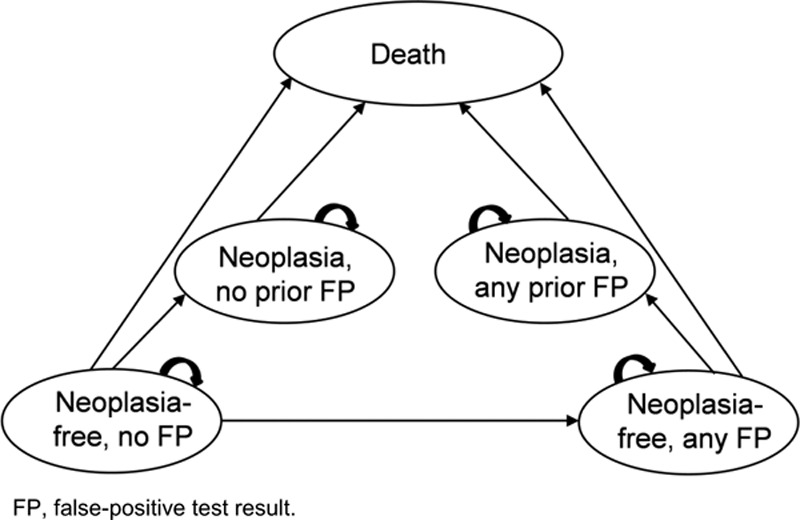
. Structure of the Markov model.

We used a basic state-transition Markov model with annual cycles to estimate the cumulative number of false-positive test results per 100 000 50-year-old persons. The model simulates a hypothetical cohort of people from age 20 to death in which the prevalence of colorectal neoplasia is increasing with age. Within a certain age range, the cohort was assumed to be exposed to a non-invasive CRC screening test applied at a regular screening interval. The structure of the Markov model is illustrated in Fig. 1. Given our research question, the states of the Markov model focussed on whether or not a person experienced any false-positive screening test result. ‘False-positive’ means that the screening test was positive although the person did not have colorectal neoplasia. At each screening round, neoplasia-free persons eligible for screening had a chance of receiving a false-positive test result, with the proportion depending on the specificity of the screening test. The model also included health states to consider initially neoplasia-free persons (with or without any prior false-positive screening test result) who developed colorectal neoplasia afterwards.

### Parameter estimates and assumptions

The cohort was assumed to develop colorectal neoplasia according to age-specific incidence rates estimated based on data of the colonoscopy arm (including 1420 participants) of the colonoscopy trial. The colonoscopy trial is a randomized controlled trial comparing colonoscopy and CT colonography in a population-based CRC screening program [6]. To consider all-cause mortality in the cohort, we used age-specific probabilities of death derived from the Dutch Central Bureau for Statistics [7]. As regards screening, we varied the starting age of screening, the screening interval and the false-positive rate (i.e. one minus specificity) of a single screening test, resulting in 11 different screening scenarios (Table 1). Given the conceptual focus of our analyses, we assumed perfect adherence among those eligible for screening.

**Table 1.**
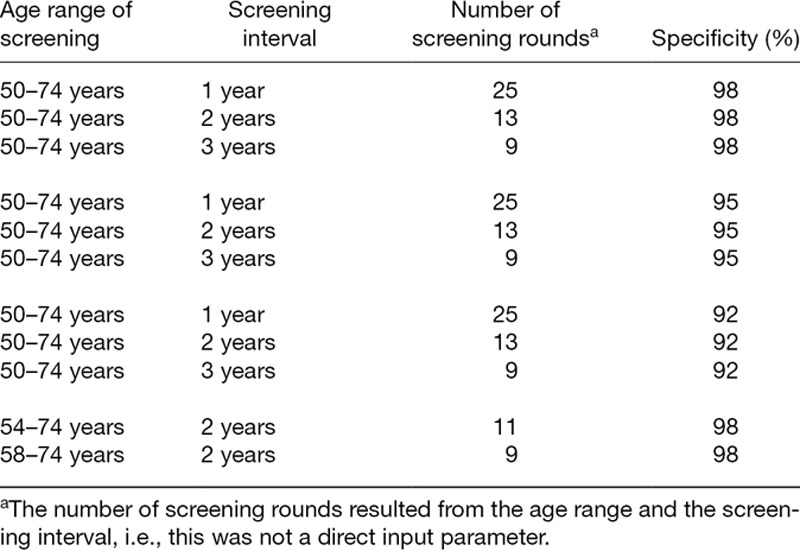
Description of the various screening scenarios: input parameter values used for the starting age of screening, the screening interval and the specificity of the screening test

In the base-case analysis, we assumed conditional independence of sequential testing. This means that the same false-positive rate (e.g. 5%, corresponding to a specificity of 95%) was applied to all persons, irrespective of whether they performed the test for the first time or had already received a true-negative test result at a previous round. True-negative means that a neoplasia-free person receives a negative test result. However, persons with a series of consecutive true-negative tests may represent a fraction of the cohort that is less prone to testing false-positive. This hypothesis has been generated by studies showing a decline in the false-positive rate between the first and the second screening round [8,9]. To take this potential deviation from the assumption of conditional independence into account, we performed sensitivity analyses for all scenarios where we decreased the false-positive rate by 1% among persons with one or more prior true-negative test results.

### Model outcome

To calculate the cumulative number of persons with any false-positive test result (cumFP) by age, we took into account all persons who ever received a false-positive test result. The cumFP thus also includes persons with a prior false-positive test result who developed colorectal lesions afterwards. We determined the cumFP by age relative to 100 000 persons neoplasia-free at age 50 for various screening scenarios. In addition, we determined the cumFP by age per 100 000 persons neoplasia-free at age 50 for the various scenarios.

## Results

**Fig. 2.**
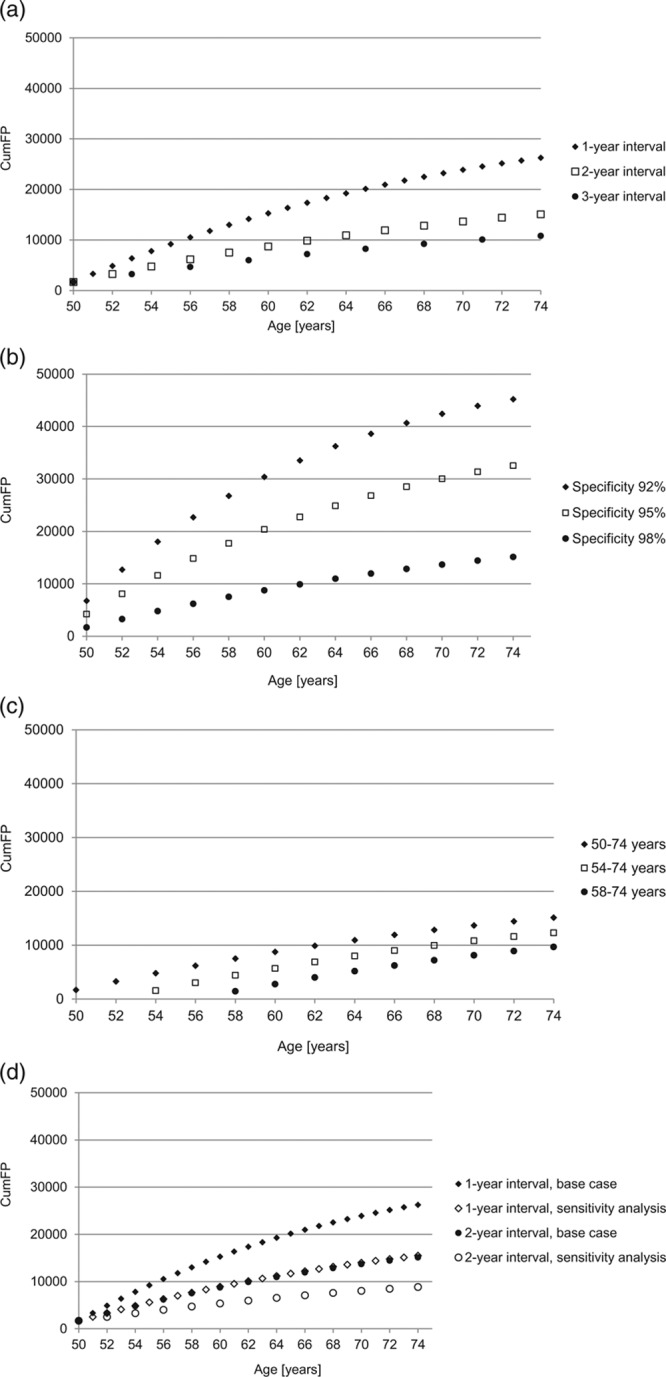
(a) Cumulative number of false-positive test results (cumFP) per 100 000 50-year-old persons for screening in the age range 50–74 years based on a test with a specificity of 98%: variation of the screening interval. (b) Cumulative number of false-positive test results (cumFP) per 100 000 50-year-old persons for screening in the age range 50–74 years with a test used at a 2-year screening interval: variation of the test specificity. (c) Cumulative number of false-positive test results (cumFP) per 100 000 50-year-old persons for screening based on a test with a specificity of 98% used at a 2-year interval: variation of the starting age of screening. (d) Cumulative number of false-positive test results (cumFP) per 100 000 50-year-old persons for screening in the age range 50–74 years based on a test with a specificity of 98% used at a 1- or 2-year interval: sensitivity analysis regarding potential deviation from the assumption of conditional independence of sequential testing.

Table 2 shows the cumFP per 100 000 50-year-old persons for all screening scenarios in the age range 50–74 years. Figure 2a illustrates these model results for a test with a specificity of 98% that is used from 50 to 74 years at an interval of 1, 2, or 3 years. At age 74, the cumFP was 26 260 (1-year interval), 15 102 (2-year interval), and 10 819 (3-year interval), respectively. Compared to the 2-year interval, the cumFP at age 74 was thus 74% higher for the 1-year interval and 28% lower for the 3-year interval.

**Table 2.**
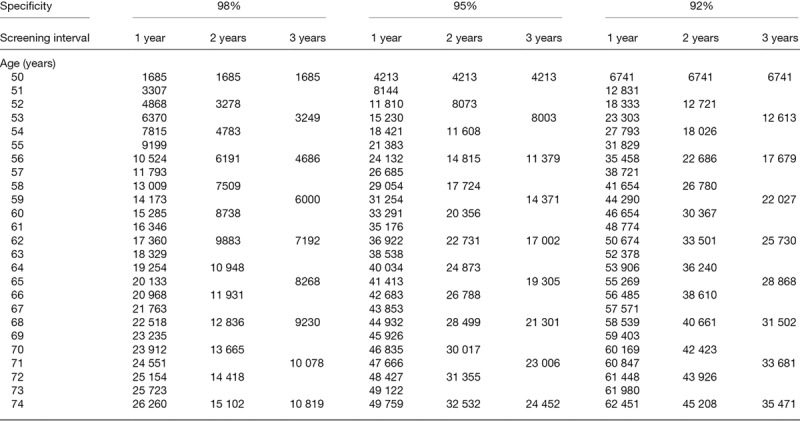
Cumulative number of false-positive test results by age per 100 000 50-year-old persons for various screening scenarios in the age range 50–74 years

Figure 2b illustrates the effect of varying specificity for a test that is used from 50 to 74 years at an interval of 2 years. For a test with a specificity of 98%, the cumFP at age 74 was 15 102 and thus 54% lower as compared to a test with a specificity of 95%. For a test with a specificity of 92%, the cumFP at age 74 was 45 208 and thus 39% higher as compared to a test with a specificity of 95%. For a test with a specificity of 92% used at a 1-year interval, the cumFP reached levels above 60 000 from the age of 70, for example, 62 451 at age 74, the maximum cumFP of all scenarios (Table 1).

Figure 2c shows the effect of shifting the starting age of screening from 50 years to 54 or 58 years for a test with a specificity of 98% used at a 2-year interval. At age 74, the cumFP was 12 312 for the scenario where screening starts at age 54 and thus 18% lower as compared to the scenario where screening starts at age 50. When screening starts at age 58 the cumFP at age 74 was 9661 and thus 22% lower compared to starting at age 54.

Figure 2d depicts the results of the sensitivity analysis where we decreased the false-positive rate among persons with a prior true-negative test result. This represented a deviation from the assumption of conditional independence of sequential testing underlying the base-case analysis. The figure shows the difference between the base-case and the sensitivity analysis exemplified for a test with a specificity of 98% that is used from 50 to 74 years at an interval of 1 or 2 years. The absolute values of the cumFP were about 40% lower from age 56 onwards (1-year interval) and from age 62 onwards (2-year interval) as compared to the base-case analysis. For the 1-year interval, the cumFP at age 74 was 15 463 in the sensitivity analysis as compared to 26 260 in the base-case analysis. For the 2-year interval, the cumFP at age 74 was 8853 as compared to 15 102 in the base-case analysis. A similar pattern was observed for a 3-year interval (see Supplementary Table 1, Supplemental digital content 1, http://links.lww.com/EJGH/A506 showing the results of this sensitivity analysis for all scenarios).

Supplementary Table 2, Supplemental digital content 2, http://links.lww.com/EJGH/A507 shows the cumFP per 100 000 persons lesion-free at age 50 (instead of using all 50-year-old persons as denominator) for all screening scenarios in the age range 50–74 years.

## Discussion

Our analysis quantitatively illustrates the importance of considering the false-positive rate in CRC screening not only for one single test but cumulatively over the screening program consisting of several rounds of testing. We found a large variation in the cumulative false-positive rate between screening strategies. For a commonly used strategy based on FIT (biennial screening from age 50 at a specificity of 98% corresponding to a cut-off level of ~20 µg haemoglobin/g faeces), our findings suggest that about 18% of persons neoplasia-free at age 50 will face a false-positive test result by age 74 if they regularly attended screening from age 50. This proportion increased to about 30% for a strategy using the test annually instead of biennially and was about 40% and above 50% for strategies using a test with a specificity of 95 and 92%, respectively, biennially.

While there are some studies reporting on false-positive findings over 2–4 rounds of FIT screening [10–13], empirical long-term evidence on the cumulative false-positive rate over several rounds of CRC screening is scarce. Zorzi *et al.* reported 12-year follow-up data on 123 347 persons to whom FIT (OC-Hemodia, cut-off level 20 µg/g faeces) was offered biennially within a population-based screening programme in north-eastern Italy [14]. They estimated that for every 1000 persons aged 50–54 years who regularly attended screening over 10 years (five rounds), about 91–98 persons without colorectal neoplasia (i.e. without CRC and adenomas) received a positive test result. Our model yielded for a scenario similar to the study by Zorzi *et al.*, that is, biennial screening from age 50–54 over five rounds, cumulative false-positive rates of 89 per 1000 assuming a specificity of 97.5% for FIT. The latter value of specificity regarding the detection of advanced and non-advanced neoplasia seems plausible for a cut-off level of 20 µg/g faeces in view of available evidence [15]. Overall, our model thus yields cumulative false-positive rates that agree well with these empirical data.

Apart from the study by Zorzi *et al.*, we found one further study that reported on the cumulative false-positive rate over five or more rounds of CRC screening. For persons starting screening at age 50, Hubbard *et al.* estimated a cumulative false-positive rate of 9% when the test was applied biennially over 10 years [16]. However, unlike in the study by Zorzi *et al.* the majority of individuals contributed only a single faecal occult blood test to the analyses, whereas more than five faecal occult blood tests were observed for only 2.7% of individuals. The estimates, which were derived by a censoring bias-adjusted discrete survival model, are thus more extrapolation rather than empirical evidence. Apart from that, comparison with the study by Zorzi *et al.* and our findings is hampered because only two ‘fair quality’ studies on the diagnostic accuracy showing divergent results regarding specificity of Hemoccult SENSA (87% vs. 96%) are available [17], so it is not clear to which level of specificity the estimates by Hubbard *et al.* refer [16,17].

While the empirical evidence regarding the cumulative FP rate is restricted to a screening period of about 10 years, screening typically spans over more than 10 years and there is variation between strategies, for example, regarding the screening interval and the cut-off level used for FIT, which determines sensitivity and specificity. Our findings are thus useful for extrapolation to longer time horizons and to scenarios for which no empirical data are available including potentially new tests. Providing the target population with information on the cumulative false-positive rate is an important component of informed decision making about screening participation. Otherwise, persons may tend to underestimate the high likelihood that participation in an initially non-invasive screening program ultimately leads to colonoscopy and, for example, not be aware of the relevance of the screening interval in this regard. Actually, the scenarios for which our model showed cumulative false-positive rates of 40% and more represent strategies that are currently recommended in some countries such as in Germany [18]. In view of the high impact of specificity on the cumulative false-positive rate, our findings underline the importance of monitoring the cut-off level of FIT in ongoing screening programs given that colonoscopy resources are typically limited. Our findings are also relevant to public health decision-makers in the process of planning CRC screening programs. Intuitively, one may assume that FIT screening leads to fewer unnecessary colonoscopies compared to colonoscopy screening. However, out of 100 000 persons lesion-free at age 50, about 54% are still lesion-free at age 74, that is, if all 100 000 persons underwent once-only colonoscopy, about 54% are equally unnecessary as those colonoscopies done in lesion-free persons with (false) positive FIT result. This proportion is rather close to the cumFP of certain scenarios of FIT screening, especially those using a 1-year interval or a low specificity. Still, the colonoscopy load would be higher if screening colonoscopy was offered more than once and both for FIT and screening colonoscopy, the additional burden of (partly unnecessary) surveillance colonoscopies needs to be taken into account which may differ between strategies.

We recognize both strengths and limitations to our study. We restricted the design of our analysis to components relevant to our research question, resulting in a basic Markov model. We consider this a strength because this increased clarity and transparency and reduced the number of assumptions. For example, detailing the adenoma state and modelling the progression between states which goes along with several uncertainties was not required in our context, but is required by models that aim to assess the effectiveness of CRC screening [19]. Unlike most other modelling studies on non-invasive CRC screening, we conducted sensitivity analyses regarding deviation from the assumption of conditional independence of sequential testing. Even though these sensitivity analyses did not question our conclusion regarding the relative impact of the various parameters on the cumulative false-positive rate, they demonstrate that deviation from this assumption has a non-negligible effect on absolute estimates of the cumulative false-positive rate. More empirical research is therefore needed to get further insights into patterns of conditional (in-)dependence of sequential testing that can be integrated into modelling.

In our study, we defined ‘false-positive’ as a positive test result in persons free of CRC and any adenoma because this definition provides a conservative estimate of the cumulative false-positive rate. We did not stratify our analyses by sex although there may be differences in the cumulative false-positive rate between men and women. To conduct sex-specific analyses, both differences in adenoma prevalence and potential differences in test specificity between men and women are important to consider. Regarding the latter, some studies suggested a lower specificity of FIT in men than in women, particularly at lower cut-off levels, but evidence is limited [20,21]. Given that the higher adenoma prevalence and lower test specificity in men may compensate each other, resulting in false-positive rates similar to women, we feel that sex-specific analyses should only be conducted when more evidence on sex-specific test performance is available.

In conclusion, our findings demonstrate the magnitude of the cumulative false-positive rate in CRC screening and show its large variation between screening strategies, which is relevant to both informed decision making and for adequate resource planning. While we provide model-based estimates on the cumulative false-positive rate, future screening studies should also consider longitudinal analyses on the false-positive rate to extend empirical evidence in this field.

## Acknowledgements

There was no external funding source.

All authors had access to all data and take responsibility for the integrity of the data. Study concept and design: U.H. and V.M.H.C. Statistical analysis: V.M.H.C. Interpretation of data: U.H. and V.M.H.C. Drafting of the manuscript: U.H. Critical revision of the manuscript for important intellectual content: V.M.H.C. Final approval of the final manuscript version: U.H. and V.M.H.C.

Ethics approval and consent to participate: Not applicable - this is a theoretical work.

The manuscript does not contain any individual person’s data in any form (including individual details, images or videos).

The Markov model used in the current study is available from the corresponding author on request.

## Conflicts of interest

There are no conflicts of interest.

## Supplementary Material
