# Supplementary material for: The cumulative false-positive rate in colorectal cancer screening: a Markov analysis
Source: Eur J Gastroenterol Hepatol. 2020 Mar 4;32(5):575–80. doi: 10.1097/MEG.0000000000001669 (PMC7147410; doi:10.1097/MEG.0000000000001669)
Supplement: Supplementary file 1 [file ejgh-32-575-s001.pdf]

Supplementary Table 1: Cumulative number of false-positive test results by age per 100 000 persons neoplasia-free at age 50 for various screening scenarios in the age range 50–74 years: sensitivity analysis regarding the assumption of conditional independence of sequential testing

Specificity	98%			95%			92%		
Screening interval	1 year	2 years	3 years	1 year	2 years	3 years	1 year	2 years	3 years
Age [years]									
50	1 685	1 685	1 685	4 213	4 213	4 213	6 741	6 741	6 741
51	2 512			7 397			12 131		
52	3 316	2 497		10 396	7 339		17 048	12 034	
53	4 098		2 483	13 222		7 283	21 535		11 938
54	4 857	3 273		15 885	10 232		25 629	16 775	
55	5 592			18 382			29 347		
56	6 302	4 005	3 223	20 722	12 881	10 045	32 722	20 982	16 466
57	6 989			22 917			35 787		
58	7 654	4 698		24 975	15 309		38 570	24 715	
59	8 297		3 907	26 904		12 517	41 097		20 392
60	8 917	5 350		28 709	17 528		43 385	28 019	
61	9 515			30 396			45 457		
62	10 092	5 964	4 533	31 973	19 549	14 713	47 333	30 934	23 768
63	10 648			33 448			49 033		
64	11 185	6 541		34 827	21 391		50 571	33 506	
65	11 700		5 104	36 111		16 654	51 958		26 658
66	12 195	7 078		37 305	23 054		53 207	35 755	
67	12 670			38 417			54 334		
68	13 126	7 578	5 620	39 452	24 555	18 353	55 349	37 721	29 108
69	13 564			40 415			56 264		
70	13 981	8 040		41 304	25 900		57 082	39 427	
71	14 379		6 079	42 126		19 819	57 815		31 155
72	14 758	8 464		42 885	27 097		58 470	40 896	
73	15 119			43 587			59 056		
74	15 463	8 853	6 484	44 235	28 161	21 075	59 580	42 162	32 853
