# Supplementary material for: The cumulative false-positive rate in colorectal cancer screening: a Markov analysis
Source: Eur J Gastroenterol Hepatol. 2020 Mar 4;32(5):575–80. doi: 10.1097/MEG.0000000000001669 (PMC7147410; doi:10.1097/MEG.0000000000001669)
Supplement: Supplementary file 2 [file ejgh-32-575-s002.pdf]

Supplementary Table 2: Cumulative number of false-positive test results by age per 100 000 persons neoplasia-free at age 50 for various screening scenarios in the age range 50–74 years.

Specificity	98%			95%			92%		
Screening interval	1 year	2 years	3 years	1 year	2 years	3 years	1 year	2 years	3 years
Age [years]									
50	2 000	2 000	2 000	5 000	5 000	5 000	8 000	8 000	8 000
51	3 925			9 664			15 227		
52	5 777	3 890		14 015	9 580		21 756	15 097	
53	7 559		3 856	18 074		9 497	27 654		14 968
54	9 274	5 676		21 860	13 775		32 982	21 392	
55	10 916			25 375			37 773		
56	12 489	7 347	5 561	28 638	17 581	13 503	42 079	26 922	20 980
57	13 996			31 667			45 951		
58	15 438	8 911		34 479	21 034		49 431	31 781	
59	16 819		7 120	37 090		17 055	52 560		26 140
60	18 139	10 370		39 507	24 157		55 366	36 037	
61	19 399			41 744			57 881		
62	20 602	11 729	8 535	43 816	26 975	20 177	60 136	39 757	30 535
63	21 751			45 734			62 158		
64	22 849	12 993		47 509	29 518		63 971	43 006	
65	23 892		9 812	49 145		22 910	65 589		34 258
66	24 884	14 158		50 653	31 790		67 032	45 819	
67	25 826			52 042			68 320		
68	26 722	15 233	10 953	53 322	33 821	25 278	69 470	48 253	37 384
69	27 574			54 501			70 495		
70	28 377	16 216		55 580	35 622		71 404	50 345	
71	29 135		11 959	56 566		27 302	72 208		39 970
72	29 851	17 110		57 469	37 209		72 921	52 128	
73	30 526			58 295			73 553		
74	31 163	17 922	12 840	59 050	38 607	29 017	74 112	53 650	42 094
